# Maresin 2, a Specialized Pro-Resolution Lipid Mediator, Reduces Pain and Inflammation Induced by *Bothrops jararaca* Venom in Mice

**DOI:** 10.3390/toxins17080367

**Published:** 2025-07-25

**Authors:** Kassyo L. S. Dantas, Beatriz H. S. Bianchini, Matheus D. V. da Silva, Maiara Piva, Joice M. da Cunha, Janaina M. Zanoveli, Fernanda C. Cardoso, Fabiana T. M. C. Vicentini, Camila R. Ferraz, Patricia B. Clissa, Rubia Casagrande, Waldiceu A. Verri

**Affiliations:** 1 Laboratory of Pain, Inflammation, Neuropathy, and Cancer, Department of Immunology, Parasitology and General Pathology, Londrina State University, Londrina 86057-970, PR, Brazil; 2 Department of Pharmacology, Federal University of Paraná, Curitiba 81531-980, PR, Brazil; 3 Institute of Molecular Biosciences, The University of Queensland, St Lucia, QLD 4072, Australia; 4 School of Pharmaceutical Sciences of Ribeirao Preto, University of Sao Paulo, Ribeirao Preto 14040-903, SP, Brazil; 5 Laboratory of Immunopathology, Butantan Institute, São Paulo 05503-900, SP, Brazil; 6 Department of Pharmaceutical Sciences, Center of Health Sciences, Londrina State University, Londrina 86039-440, PR, Brazil; rubiacasa@uel.br

**Keywords:** maresin 2, *Bothrops jararaca* venom, pain, inflammation, hyperalgesia, nitric oxide, superoxide anion, neutrophil, macrophage, hemorrhage

## Abstract

The venom of *Bothrops jararaca* (BjV) induces intense and prolonged pain, which is not alleviated by antivenom, along with hemorrhage and inflammation. In this study, we investigated the effects of the specialized pro-resolving lipid mediator (SPM) maresin 2 (MaR2) in a murine model of BjV-evoked pain and inflammation. Mice received a single intraperitoneal (i.p.) injection of MaR2 30 min before the intraplantar BjV injection. MaR2 treatment significantly attenuated mechanical (electronic aesthesiometer) and thermal (hot plate) hyperalgesia in a dose-dependent manner. Additionally, MaR2 restored the balance for the hind-paw static weight distribution. When BjV (0.01, 0.1, and 1 μg) stimulus was administered intraperitoneally, pre-treatment with MaR2 (0.3, 1, or 3 ng) ameliorated mechanical and thermal hyperalgesia in a dose-dependent manner. Moreover, MaR2 (3 ng) effectively reduced the levels of myeloperoxidase activity and cytokines (TNF-α, IL-1β, and IL-6) and superoxide anion (O_2_^•−^) production induced by intraplantar injection of BjV while enhancing total antioxidant levels (ABTS scavenging). For the peritonitis model induced by BjV, MaR2 pretreatment decreased leukocyte recruitment, hemorrhage, nitric oxide (NO), and O_2_^•−^ generation and gp91phox and inducible nitric oxide synthase (iNOS) mRNA expression. In conclusion, this study presents the first evidence that MaR2 effectively mitigated BjV-induced pain, hemorrhage, and inflammation.

## 1. Introduction

In Brazil, the snake species *Bothrops jararaca* (commonly known as “jararaca”) is responsible for the majority of venomous snakebites [1]. The clinical symptoms induced by the *Bothrops jararaca* venom (BjV) can be diverse and severe, including swelling, bruises, blisters, abscesses, tissue necrosis, and intense pain. These symptoms can escalate, potentially leading to extensive tissue damage, physical disability, or even amputation [2].

Understanding the bothropic venom pathogenesis is challenging due to the complex mixture of pharmacologically active peptides and proteins found in its composition that can initiate the wide range of symptoms observed in the victims; although, it has been described that the local manifestations are mainly attributed to serin proteases, metalloproteases, and phospholipase A2 effects [3,4,5,6,7]. Leukocyte migration in response to venom leads to the further release of pro-inflammatory cytokines and an excessive production of reactive oxygen species (ROS), such as superoxide anion (O_2_^•−^) and nitric oxide (NO) [8,9]. Along with the cellular debris from phagocyte activity, the production of ROS can cause damage to cellular components like lipids, proteins, and nucleic acids, leading to cell death and tissue injury, thus contributing to the progression of inflammation [10].

Treatment for the *Bothrops* snake envenomation is the intravenous injection of bothropic antivenom [11], which is composed of immunoglobulins or immunoglobulin fragments obtained from the plasma of animals immunized with venom, and acts by neutralizing its systemic actions but presents low effectiveness against the injury site symptoms [12,13]. However, the prolonged and intense pain and inflammation caused by BjV cannot be reversed by the antivenom [3,14].

In clinical practice, pain management and inflammation control in patients envenomed by Bothrops species is typically addressed not only with antivenom therapy, but also with adjunctive pharmacological agents. Non-opioid analgesics such as dipyrone and paracetamol are commonly used to relieve pain, showing efficacy in inflammatory pain models despite their weak anti-inflammatory profiles [15]. Corticosteroids may be used selectively to reduce inflammation, particularly in complex or severe cases of snakebites [16,17]. However, corticoids are immunosuppressive, which may be a problem since there is an incidence ranging from 9% to 77% of bacterial infection in snakebite wounds [18]. NSAIDs like acetylsalicylic acid (aspirin) are contraindicated in these patients because of their antiplatelet effect, which could worsen venom-induced hemorrhage, as observed in other hemorrhagic conditions like dengue fever [19]. Thus, careful selection of co-analgesic and co-anti-inflammatory therapy is essential to avoid aggravating the hemorrhagic and inflammatory effects of the venom. However, there is still need of improving the management of pain and inflammation caused by snakebites [3,14].

Specialized pro-resolution lipid mediators (SPMs), such as lipoxins, resolvins, protectins, and maresins [20] represent a promising therapeutic group of molecules. In addition to their pro-resolution effects, their activities generally include anti-inflammatory and analgesic effects without inducing immunosuppression [21,22,23,24]. Maresins are the latest group of SPMs to be described, and its main members are maresin 1 (MaR1) and maresin 2 (MaR2) [23,24,25,26].

MaR2 reduces the recruitment of neutrophils in zymosan peritonitis [27] and induces tissue repair via pro-resolution activities such as efferocytosis [27,28,29]. MaR2 has also shown analgesic properties in studies using models of lipopolysaccharides (LPS)-induced inflammation [30] and neuropathic pain [31]. MaR2 reduced the transient receptor potential vanilloid 1 (TRPV1)- and transient receptor potential ankyrin 1 (TRPA1)-dependent activation on nociceptor neurons in calcium quantitation and behavior assays [30]. It markedly reduced peripheral inflammation, as observed by a decrease in the recruitment of CD45+F4/80-Ly6G+ neutrophils and CD45+F4/80+Ly6C+ macrophages. The downmodulation of phagocyte recruitment was explained by targeting the production of pro-inflammatory tumor necrosis factor alpha (TNF-α), interleukins, chemokines, and neuropeptides [30]. This suggests MaR2’s therapeutic potential in conditions in which recruited phagocytes (e.g., neutrophils and macrophages) have a pathological role.

The prior evidence of the activity of MaR2 in models of LPS paw inflammation neuropathic pain and zymosan peritonitis [27,30,31] suggests that this SPM could present similar activity in other disease models, which would broaden its range of potential therapeutic applications. However, to the best of our knowledge, there is no prior evidence investigating the activity of MaR2 or other SPM against venom-induced pain or inflammation. Therefore, the present study investigated the activity and mechanisms of MaR2 against BjV pathology in mice focusing on pain and inflammation.

## 2. Results

### 2.1. BjV Induces Dose- and Time-Dependent Hyperalgesia That Is Amenable by MaR2 Treatment

**Figure 1:**
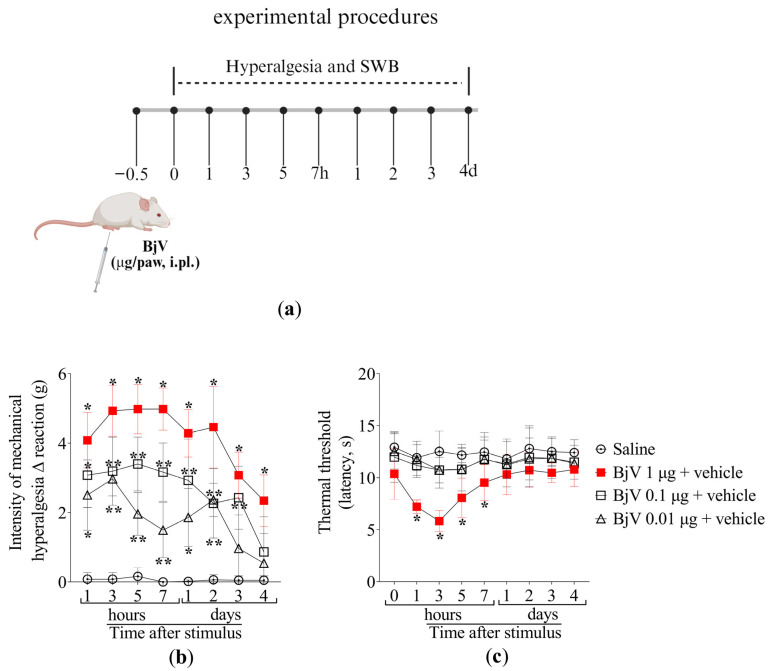
The intraplantar (i.pl.) injection of BjV induces mechanical and thermal hyperalgesia. (**a**) Schematic protocol of BjV dose–response curves of hyperalgesia. (**b**) Mechanical and (**c**) thermal hyperalgesia were evaluated after 1, 3, 5, and 7 h, and daily for 4 days after BjV injection (1, 0.1 e 0.01 μg/paw, i.pl.) applying the electronic von Frey and hot plate tests, respectively. Results are presented as Δ means ± SD (**b**,**c**); n = 6 mice per group (* *p* < 0.05 vs. saline; ** *p* < 0.05 vs. saline and BjV (1 µg) [two-way ANOVA followed by Tukey]).

BjV induced mechanical and thermal hyperalgesia in a dose-dependent manner (Figure 1a–c). Mechanical hyperalgesia lasted up to 4 days and thermal hyperalgesia persisted up to 7 h upon a single 1 μg injection of BjV (Figure 1b,c). Therefore, the BjV dose of 1 μg was selected for the following experiments, aiming to investigate the analgesic effect of MaR2.

**Figure 2:**
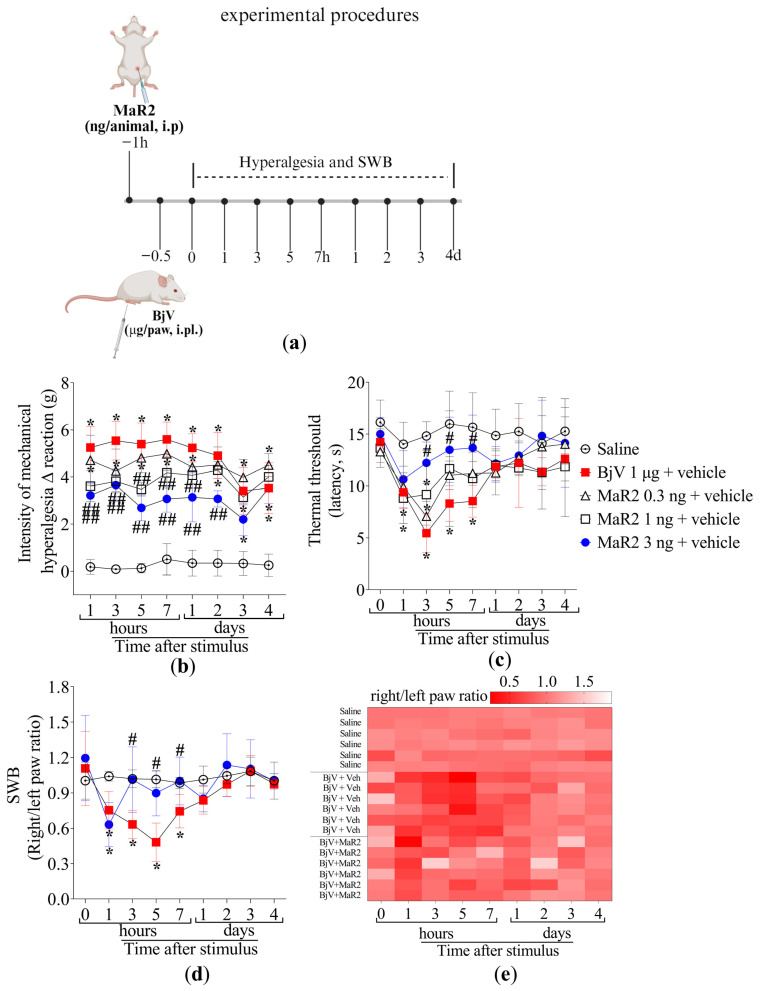
MaR2 reduces inflammatory pain induced by BjV. (**a**) Scheme of treatment protocols. (**b**,**c**) MaR2 dose–response (0.3, 1 e 3 ng/animal, i.p.) 30 min before BjV injection (1 μg/paw, i.pl.); (**b**) mechanical and (**c**) thermal hyperalgesia were evaluated applying the electronic von Frey and hot plate tests, respectively. Results are presented as Δ means ± SD (**b**,**c**); n = 6 mice per group (* *p* < 0.05 vs. saline; # *p* < 0.05 vs. BjV; ## *p* < 0.05 vs. saline and BjV [two-way ANOVA followed by Tukey]). (**d**) SWB was applied to assess non-evoked nociceptive behavior. (**e**) The heatmap shows the ratio of right to left hind paws of each mouse in different time-points, with intense shades of red demonstrating an imbalanced right/left hind paw ratio (indicating pain), while lighter shades of red towards white demonstrate a balanced right/left hind paw ratio (indicating diminishing pain or lack of it when it is white). Results are presented as the ratio of right to left hind paws of each mouse; n = 6 mice per group (* *p* < 0.05 vs. saline; # *p* < 0.05 vs. BjV [two-way ANOVA followed by Tukey]).

Mice were treated intraperitoneally (i.p.) with 0.3, 1, or 3 ng per cavity of MaR2 30 min prior to BjV injection, as described in Figure 2a. We selected the i.p. route because it is widely used in proof-of-concept studies aiming to first demonstrate the full potential activity of a drug. These proof-of-concept studies allow others to follow up by testing other routes of administration or developing pharmaceutical forms attending to specific needs. The saline group did not exhibit hyperalgesia in both electronic von Frey (mechanical hyperalgesia) and hot plate (thermal hyperalgesia) assays. BjV induced mechanical hyperalgesia at all evaluated time-points, while thermal hyperalgesia was observed as far as the 7th hour post-injection (Figure 2b,c). The 0.3 ng dose of MaR2 did not attenuate BjV-induced mechanical or thermal hyperalgesia (Figure 2b,c). Treatment with 1 ng of MaR2 partially reduced mechanical hyperalgesia on the first day, whereas the 3 ng dose significantly attenuated mechanical hyperalgesia up to 48 h after BjV injection (Figure 2b). Thermal hyperalgesia was inhibited from the 3rd to the 7th hour (Figure 2c). Thus, the 3 ng dose of MaR2 was chosen for subsequent experiments. It is important to mention that Figure 1c and Figure 2c are showing the thermal threshold values; thus, the BjV diminishes the threshold to trigger a nociceptive behavior caused by thermal stimulation. This result is observed as a reduction in the thermal threshold to elicit the nociceptive behavior of removing the paw from the thermal stimulus.

To assess hyperalgesia using a non-evoked approach, changes in the hind-paw weight distribution were evaluated. BjV injection decreased the right- to left-paw weight distribution ratio from the 1st till 7th hour (Figure 2d–e), while pretreatment with MaR2 restored baseline values from the 3rd hour onward, indicating its analgesic effect in a non-evoked response (Figure 2d–e).

### 2.2. MaR2 Reduces Myeloperoxidase (MPO) Activity, Oxidative Stress, and Levels of TNF-α, IL-1β, and IL-6 in the Paws of BjV-Injected Mice

**Figure 3:**
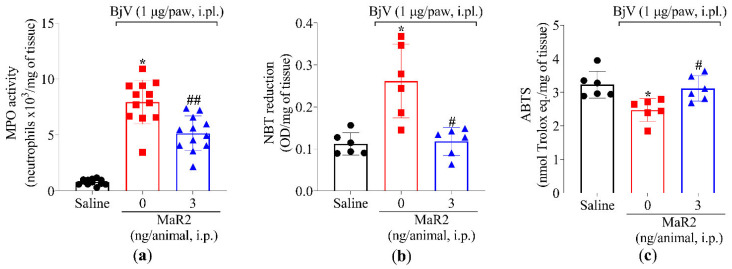
MaR2 reduces myeloperoxidase (MPO) activity and superoxide anion production and increases free radical scavenging. Mouse plantar tissue was dissected 7 h post-injection with BjV (1 μg/paw, i.pl.) to determine (**a**) MPO activity and (**b**) superoxide anion production (NBT assay) and (**c**) total antioxidant properties (scavenging of the ABTS radical). Results are presented as mean ± SD; MPO assay n = 12 mice per experimental group; NBT and ABTS assay n = 6 mice per group (* *p* < 0.05 vs. saline; # *p* < 0.05 vs. BjV; ## *p* < 0.05 vs. saline and BjV [one-way ANOVA followed by Tukey]).

During the early stages of the acute inflammatory response, polymorphonuclear leukocytes (PMNs), primarily neutrophils, are recruited to post-capillary venules to initiate the phagocytosis of microorganisms and cellular debris [32]. However, excessive PMNs activation in inflamed tissue can exacerbate inflammation, leading to tissue damage [33]. Neutrophil and macrophage recruitment can be assessed by MPO activity, an enzyme expressed by these phagocytes [34,35]. The i.pl. injection of BjV significantly increased MPO activity in the plantar tissue, an effect that was attenuated by MaR2 treatment (Figure 3a). BjV also induced oxidative stress, as evidenced by an increase in superoxide anion production (assessed via the nitroblue tetrazolium [NBT] assay, Figure 3b), and a reduction in the antioxidant activity of the 2,2′-azino-bis(3-ethylbenzothiazoline-6-sulfonic acid (ABTS) cationic radical scavenging assay (Figure 3c). Both effects were reversed by MaR2 (Figure 3b,c).

**Figure 4:**
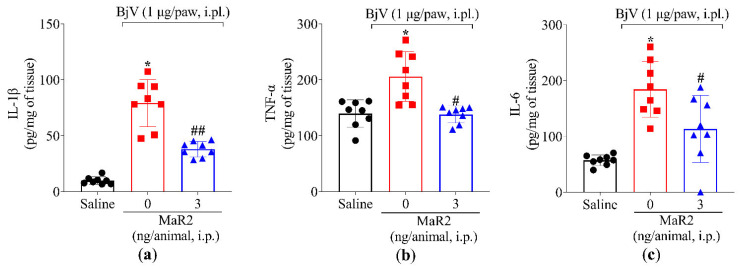
MaR2 downregulates pro-inflammatory cytokine levels. Following the protocol of Figure 1a, mouse plantar tissue was dissected 7 h post-injection with BjV (1 μg/paw, i.pl.) to determine the production of (**a**) IL-1*β*, (**b**) TNF*α*, and (**c**) IL-6 by ELISA. Results are presented as mean ± SD; n = 8 per group (* *p* < 0.05 vs. saline; # *p* < 0.05 vs. BjV; ## *p* < 0.05 vs. saline and BjV [one-way ANOVA followed by Tukey]).

Furthermore, MaR2 inhibited the BjV-increased production of tumor necrosis factor-alpha (TNF-α), interleukin-1β (IL-1β), and interleukin-6 (IL-6) (Figure 4a–c). These findings demonstrate that, under the present experimental conditions, BjV injection promotes leukocyte recruitment (characterized by MPO activity) as well as oxidative stress and pro-inflammatory cytokine production. In response, MaR2 effectively mitigated these pathologic alterations, which partially explains its analgesic effects.

### 2.3. MaR2 Inhibits Leukocyte Recruitment, Hemorrhage, and Oxidative Stress in Peritonitis Induced by BjV

#### 2.3.1. MaR2 Inhibits Leukocyte Recruitment

**Figure 5:**
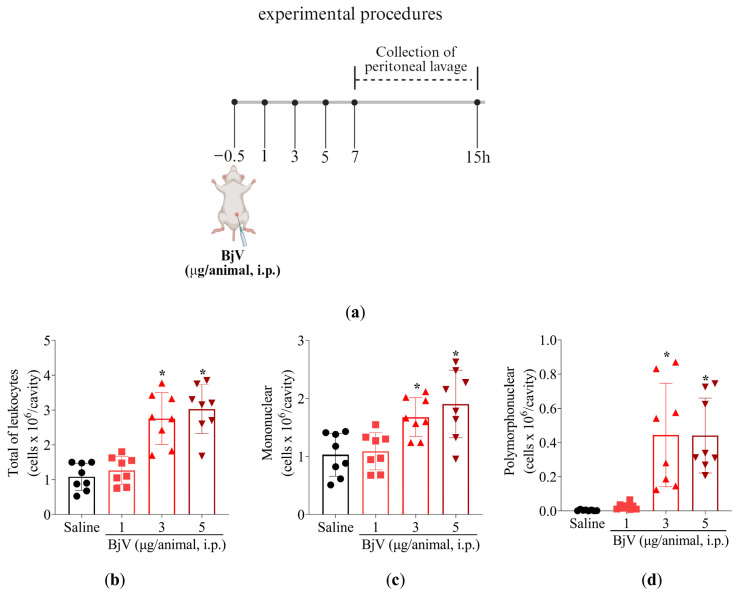
BjV intraperitoneal injection induces leukocyte recruitment. (**a**) Schematic protocol. (**b**–**d**) BjV dose-dependent (1, 3, and 5 μg/cavity, i.p.) effect on leukocyte recruitment at 15 h post-peritoneal injection. Results are presented as mean ± SD; n = 8 mice per group (* *p* < 0.05 vs. saline [one-way ANOVA followed by Tukey]).

**Figure 6:**
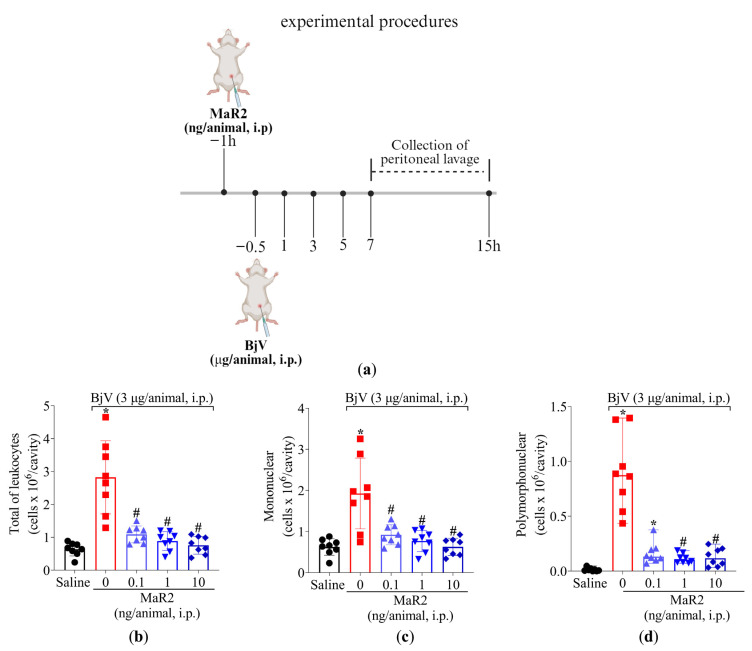
MaR2 inhibits peritoneal leukocyte recruitment induced by BjV. (**a**) Schematic protocol of MaR2 dose–response treatment against BjV recruitment of leukocytes. (**b**–**d**) A dose–response was conducted to determine MaR2’s optimal dose (0.1, 1, and 10 ng/cavity, i.p., injected 30 min before BjV induction). Results are presented as mean ± SD (**b**,**c**) and median with range (**d**); n = 8 mice per group (* *p* < 0.05 vs. saline; # *p* < 0.05 vs. BjV) for total leukocyte (**b**) and mononuclear (**c**) cells assessment [one-way ANOVA followed by Tukey—parametric data]; and polymorphonuclear (**d**) cells [Kruskal–Wallis followed by Dunn’s—non-parametric data]).

Initially, titrated doses of BjV were tested (1, 3, and 5 μg/cavity) to determine the ideal stimulus intensity (Figure 5a–d). This approach allows better assessment of which leukocytes were being recruited upon BjV stimulus (Figure 5) and MaR2 treatment (Figure 6). Peritoneal cell washes were collected 15 h after BjV injection. BjV injection increased total leukocyte, mononuclear cells, and neutrophil recruitment with a similar effect between the 3 and 5 μg doses of BjV (Figure 5b–d). Thus, 3 μg of BjV was selected for the following experiments.

Following treatment protocol described in Figure 6a, MaR2 (0.1, 1, and 10 ng/cavity) i.p. treatment 30 min before BjV injection effectively reduced total leukocytes recruitment, mononuclear cells, and neutrophils. MaR2’s maximum activity was observed with 1 and 10 ng, which reduced all parameters (Figure 6b–d). Therefore, the 1 ng/cavity dose of MaR2 was selected for the following experiments.

#### 2.3.2. MaR2 Inhibits BjV-Induced Hemorrhage

**Figure 7:**
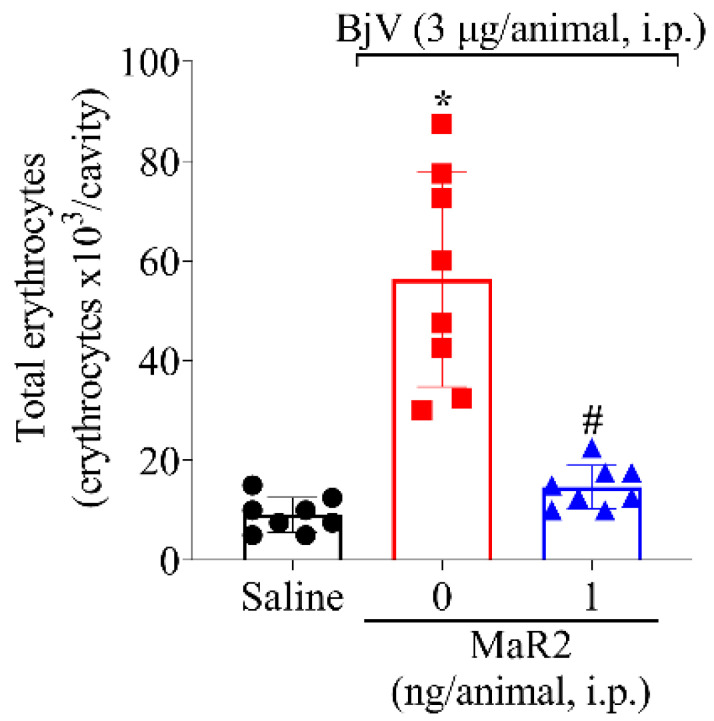
MaR2 inhibits BjV-induced hemorrhage. Peritoneal cell wash was collected 15 h after BjV injection (3 μg/cavity, i.p.) to analyze total erythrocyte count. Results are presented as mean + SD; n = 8 mice per group (* *p* < 0.05 vs. saline; # *p* < 0.05 vs. BjV [one-way ANOVA followed by Tukey]).

Local hemorrhage is a result of bothropic envenomation, and it is caused by the activity of metalloproteinases present in the BjV [36,37,38]. The intraperitoneal injection of BjV could mimic this important envenomation parameter. BjV induced an increase in erythrocytes in the peritoneal cavity and MaR2 reduced this effect (Figure 7).

#### 2.3.3. MaR2 Inhibits Leukocyte Activity and Oxidative Stress Caused by BjV

**Figure 8:**
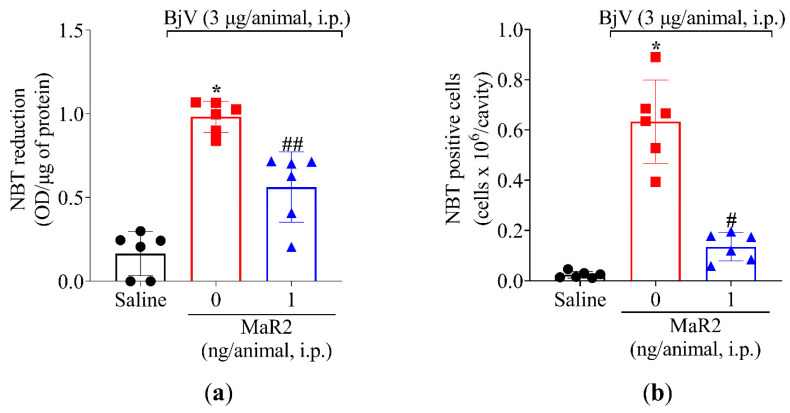
MaR2 reduces superoxide anion production in BjV-induced peritonitis model. (**a**) The effect of MaR2 on superoxide anion production (NBT assay) and (**b**) NBT-positive cell counts on peritoneal exudate were evaluated 15 h post BjV stimulus. NBT-positive cell count was performed with 40× magnification using a conventional light microscope. NBT and formazan: n = 6 mice per group (* *p* < 0.05 vs. saline; # *p* < 0.05 vs. BjV; ## *p* < 0.05 vs. saline and BjV [one-way ANOVA followed by Tukey]). Results are presented as mean ± SD.

**Figure 9:**
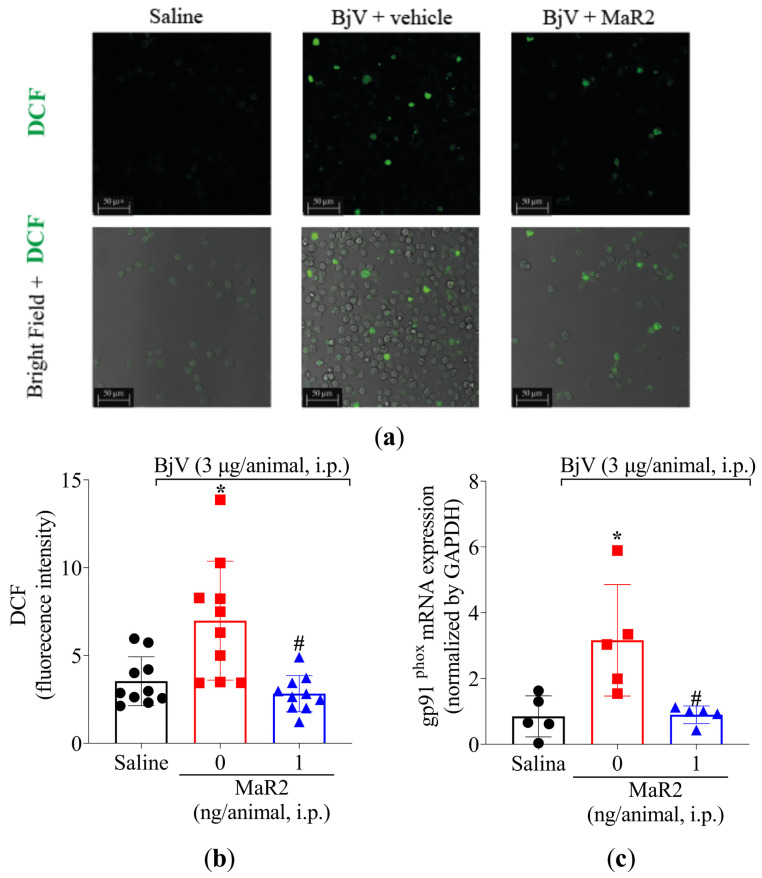
MaR2 reduces intracellular ROS and gp91phox mRNA expression triggered by BjV peritonitis. (**a**,**b**) Levels of intracellular ROS were detected using H2-DCF probe on brightfield and 488 nm channel with 63x magnification on confocal microscope. Size bars are indicative of 50 μm. DCF assay: n = 10 mice per group; (* *p* < 0.05 vs. saline; # *p* < 0.05 vs. group 0 [one-way ANOVA followed by Tukey]). (**c**) gp91phox mRNA expression was evaluated 7 h after BjV peritoneal stimulus by RT-qPCR. Relative gene expression was measured using comparative 2−(ΔΔCt) with *Gapdh* as constitutive gene. gp91phox mRNA expression: n = 5 mice per group (* *p* < 0.05 vs. saline; # *p* < 0.05 vs. BjV [one-way ANOVA followed by Tukey]). Results are presented as mean ± SD.

**Figure 10:**
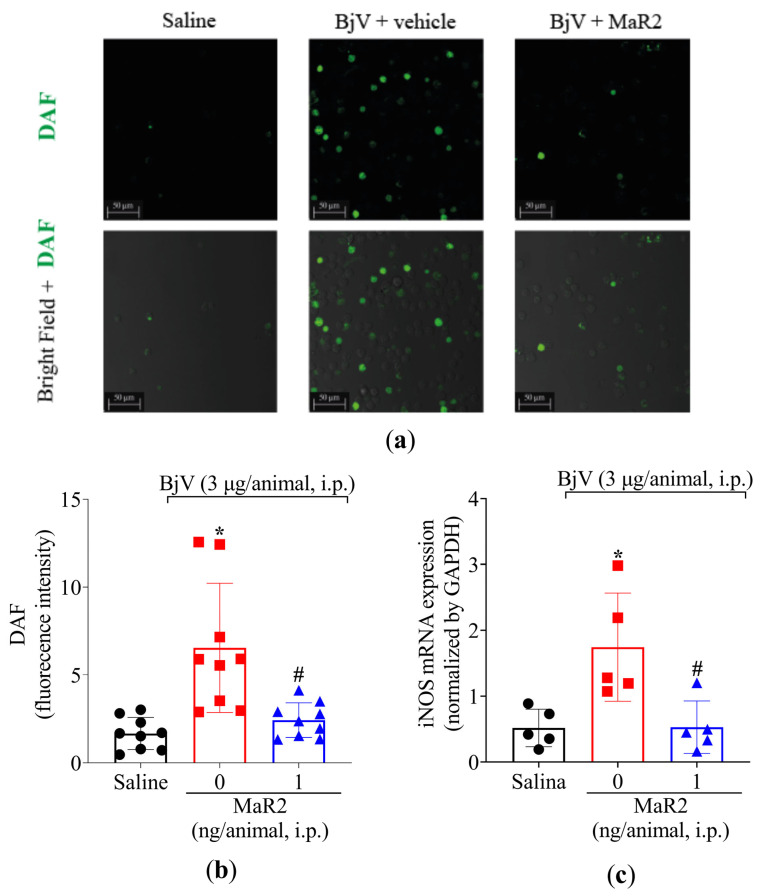
MaR2 reduces nitric oxide levels and iNOS expression on BjV-induced peritonitis. (**a**,**b**) Total NO levels were detected using the DAF-FM fluorescent probe. Size bars are 50 μm. DAF: n = 9 mice per group (* *p* < 0.05 vs. saline; # *p* < 0.05 vs. group 0 [one-way ANOVA followed by Tukey]. (**c**) iNOS mRNA expression was analyzed 7 h after BjV stimulus using 2−(ΔΔCt) comparative method and *Gapdh* as constitutive gene and RT-qPCR; n = 5 mice per group (* *p* < 0.05 vs. saline; # *p* < 0.05 vs. BjV [one-way ANOVA followed by Tukey]). Results are presented as mean ± SD.

Leukocytes recruited to the inflammatory foci can contribute to reactive oxygen species (ROS) and reactive nitrogen species (RNS) [39]. Summing up this evidence, MaR2 was able to reduce BjV oxidative stress (Figure 3) and leukocyte recruitment (Figure 3 and Figure 5). Thus, we reasoned that it would be important to assess if MaR2 could affect leukocyte production of ROS and RNS (Figure 8, Figure 9 and Figure 10). Figure 8 demonstrates that BjV stimulation leads to an increase in ROS, as detected using the superoxide anion production as observed by NBT reduction in the peritoneal exudate (Figure 8a), as well as counting NBT+ cells (Figure 8b) and DCF-DA probe (Figure 9a,b). In agreement with these data, the gp91phox mRNA expression, which is a subunit of the nicotinamide adenine dinucleotide phosphate (NADPH) oxidase subunit, was also increased (Figure 9c). MaR2 inhibited all these ROS-related parameters induced by BjV envenomation (Figure 8 and Figure 9).

In addition, BjV increased the NO production in the peritoneal exudate leukocytes as observed using the DAF-2DA probe (Figure 10a,b). This increase in NO production was parallel to an increase observed in inducible nitric oxide synthase (iNOS) mRNA expression (Figure 10c). In response, MaR2 reduced both NO production and iNOS mRNA expression triggered by BjV (Figure 10). Thus, MaR2 effectively reduced ROS and RNS production by recruited leukocytes.

## 3. Discussion

Snakebite accidents represent a serious health problem. In Brazil, 69.3% of snakebite accidents are associated with *Bothrops jararaca* [1]. Pain and inflammation are major clinical symptoms [3,14]. Numerous studies have shown the antioxidant and anti-inflammatory capacity of SPMs, especially of maresins [23,27,28,31,40]. To date, there are no reports on the activity of SPMs for snakebite-like experiments. To our knowledge, this is the first study providing evidence that MaR2 or other SPMs reduce pain and inflammation caused by BjV injection in mice.

In the present study, BjV injection caused mechanical and thermal hyperalgesia, and hind paw weight distribution imbalance, which supports that BjV causes both evoked (electronic version of von Frey filaments and hot plate) and non-evoked (static weight-bearing) nociceptive behaviors. MaR2 treatment reduced mechanical hyperalgesia, thermal hyperalgesia, and static weight-bearing distribution imbalance between hind paws caused by BjV. Although the inflammatory and painful state generated upon BjV injection are well-known, the pathological mechanisms underlying these effects are not fully investigated [3,14].

Spinal cord glial cells, such as microglia and astrocytes, play a central role in modulating hyperalgesia induced by jararhagin, a metalloproteinase found in BjV. To generate this painful state, jararhagin activates the nuclear factor kappa B (NFκB) pathway and induces the production of the pro-hyperalgesic cytokines TNF-α and IL-1β [3,4,39,40,41,42]. The analgesic activity of MaR2 in BjV inflammation was accompanied by reduction in TNF-α, IL-1β, and IL-6 levels in the paw skin, which, together with the established hyperalgesic role of these cytokines [43], supports that inhibiting such hyperalgesic cytokine production is part of the MaR2 mechanism of action. In agreement with this, MaR2 also reduced the production of TNF-α, interleukins, and chemokines after LPS-induced hyperalgesia [30].

During the acute phase of inflammation, resident cells play a crucial role in releasing pro-inflammatory cytokines and chemokines, thus orchestrating the recruitment of leukocytes to the inflamed site [44]. Previous studies have shown that BjV causes leukocyte infiltration with the predominance of neutrophils in mice muscle tissue [45], neutrophil migration in peritonitis, and increased levels of IL-6 and IL-1β in the peritoneal cavity [46,47]. BjV activity does not act directly on neutrophils to induce their recruitment, but rather it induces the production of chemotactic factors that will chemoattract neutrophils [48]. Despite the essential role of neutrophils in protecting against infections [49], these protective mechanisms can also be a source of tissue damage. Neutrophils produce ROS, such as O_2_^•−^ [50], and trigger the release of MPO, an enzyme responsible for the generation of cytotoxic oxidants, catalyzing the formation of hypochlorous acid [51,52,53,54]. Macrophages are tissue resident cells and during acute inflammation, they are among the first responders to inflammatory stimuli. They release chemoattractant molecules recruiting neutrophils. Macrophages also progressively accumulate in the inflammatory foci originating from recruited monocytes that switch their phenotype to macrophages. Macrophages use NADPH oxidase, iNOS, and MPO as mechanisms to generate superoxide anion, nitric oxide hypochlorous acid to protect against infections, but similar to neutrophils, these mechanisms also account to tissue damage [55].

MaR2 reduced superoxide anion production, endogenous antioxidant defenses (e.g., ABTS activity), and MPO activity triggered by BjV. A reduction in MPO activity can indicate diminished enzyme activity as well as an inhibition in neutrophil and macrophage recruitment [55,56,57]. Thus, to further explore MaR2 effects on enzyme inhibition and/or cellular recruitment, a BjV-induced peritonitis model was performed, allowing easy access to recruited cells [5,14]. BjV can induce leukocytes to accumulate in the peritoneal cavity, with an increase in mononuclear cells and neutrophils in the inflammatory peritoneal exudate. Evidence supports the contribution of pro-inflammatory lipid mediators [48], metalloproteinases, and phospholipases A2 present in the venom [58] to leukocyte recruitment. We also observed that BjV injection led to the recruitment of mononuclear cells and neutrophils to the peritoneal cavity and MaR2 reduced this activity. BjV-caused hemorrhage was evident in the peritoneal exudate, and MaR2 reduced such a disease phenomenon. *Bothrops jararaca* envenomation causes hemorrhage that is not restricted to the snakebite site; it can be observed systemically [59].

BjV peritonitis enabled the harvesting of peritoneal cells to assess MaR2’s activity against BjV oxidative stress. The DCF-2DA probe (for total intracellular ROS) and the DAF-2DA (for intracellular NO) probe indicated that BjV not only promoted leukocyte recruitment but also total ROS and RNS production by these cells. The NBT assay added further confirmation that BjV increases superoxide anion, a reactive oxygen species produced by cells present in the peritoneal exudate. Further supporting the detection of ROS and RNS, BjV increased the mRNA expression of gp91phox and iNOS. The gp91phox is a subunit of NADPH oxidase responsible for the conversion of molecular oxygen (O_2_) into superoxide anion (O_2_^•−^) [39], and iNOS is an enzyme involved in the significant production of nitric oxide that utilizes L-arginine as a substrate and molecular oxygen and NADPH as co-substrates [60,61]. These effects are aligned with previous data demonstrating that BjV induces microbicidal functions in peritoneal leukocytes including hydrogen peroxide and iNOS expression/NO production [61]. High doses of BjV can also increase systemic serum levels of cytokines and NO [8]. MaR2 reduced the production of total ROS, superoxide anion and NO as well as the mRNA expression of gp91phox and iNOS caused by BjV. Therefore, the targeting of ROS and RNS production represents a potential mechanism by which MaR2 reduces BjV tissue inflammation. Although assessing tissue damage is not within the aims of this study, these results suggest that MaR2 can possibly reduce such later effects caused by BjV.

MaR2 has been shown to represent an interesting SPM compared to its main family counterpart, MaR1, in terms of analgesia mechanisms. MaR1 reduces the activation of TRPV1 (transient receptor potential vanniloid 1) in nociceptive neurons as observed using electrophysiology, pharmacology, and behavior assays, explaining its analgesic effect. However, MaR1 cannot inhibit the activation of TRPA1 (transient receptor potential ankyrin 1) [62]. On the other hand, MaR2 inhibits the activation of both TRPV1 and TRPA1 [30], which represents an advantageous analgesic mechanism compared to MaR1 considering the relevance of TRPV1 and TRPA1 to pain [63,64].

**Figure 11:**
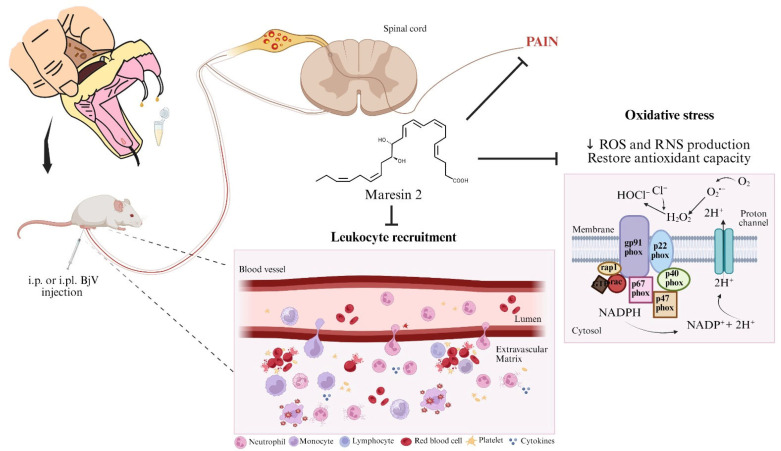
Biological activity of MaR2 against *Bothrops jararaca* venom. MaR2 reduces pain, leukocyte recruitment, cytokine levels, ROS, and RNS production induced by BjV injection in mice. The MaR2 regulation of tissue oxidative stress seemed related to gp91phox and iNOS mRNA expression. Created in BioRender. Bianchini, B. (2025) https://BioRender.com/q18p784.

In the current study, we assessed the pharmacological activity of MaR2 to limit BjV effects (Figure 11). The present study opens novel venues in the BjV field. In addition to understanding the activity of other SPMs, it is possible to develop studies assessing the endogenous resolution mechanisms of SPMs in the context of BjV. It is interesting to investigate the cellular and receptor kinetics of SPMs and their pro-resolution characteristics (e.g., cellular apoptosis, cellular phenotype shift) as well as SPM production profiles to gain deeper insight into pro-resolution mechanisms in BjV. This approach could contribute to the development of novel treatments. It is important to mention the limitations of this study. We focused on pain, inflammation, and hemorrhage; thus, other effects of BjV were not assessed, such as lethality and tissue damage. This study used a pre-treatment protocol with MaR2. Despite the prolonged effect obtained with a single treatment with MaR2, we acknowledge that post-treatment protocols and other routes of administration should be tested in future studies.

## 4. Conclusions

In conclusion, our study provides new insights into the biological activity of MaR2 in mitigating BjV-induced inflammation and pain (Figure 11). MaR2 activity was explained by a reduction in cytokines, ROS, and RNS production. The MaR2 regulation of tissue oxidative stress seems related to gene expression since gp91phox and iNOS mRNA expression were reduced. This is the first report that MaR2, an SPM, reduces BjV-induced inflammation and pain and proposes, at least in part, its targeted mechanisms. These results suggest the therapeutic potential of MaR2 to treat *Bothrops jararaca* envenomation symptoms, such as pain and inflammation, which merits further experimentation towards clinical studies.

## 5. Materials and Methods

### 5.1. Animals

A total of 336 Swiss female mice (25–30 g) were provided from Londrina’s State University (Londrina, PR, Brazil), allocated in clear standard plastic cages with free access to food and water, and submitted to 12 h light/dark cycles and constant 21 °C temperature. For all behavior experiments, mice were previously acclimated to the laboratory for at least two days during one-hour-long adaptations to the experimental cages. All steps followed IASP (International Association for the Study of Pain) guidelines and were sanctioned by the Ethics Committee of Animal Experimentation from Londrina’s State University under the protocol 19408.2019.84 with the approval number 103/2021.

### 5.2. General Experimental Procedures

All procedures were performed under protocols approved by the institutional animal care committee, with full adherence to humane endpoints. Investigators conducting the experiments were blinded to group allocation. Euthanasia was performed by isoflurane anesthesia (5% in oxygen using a precision vaporizer) followed by decapitation as a confirmatory method. Sample collection occurred only after euthanasia. No animals were excluded from statistical analysis. All efforts were made to minimize the number of animals used and their suffering. To achieve this aim, the G*Power 3.1. software was applied to calculate the n of animals per type of assay.

For the initial series of experiments, dose–response curves were performed to determine the optimal dose of *Bothrops jararaca* venom (BjV) and maresin 2 (MaR2) for subsequent studies. Female Swiss mice received intraplantar (i.pl.) injection of BjV in the right hind paw at doses of 0.01, 0.1, or 1 μg/paw (in 20 μL of saline). Mechanical and thermal hyperalgesia were assessed in the same animals at 1, 3, 5, and 7 h post-injection, and daily for the next 4 days. There were 4 groups (vehicle and three doses of BjV) with 6 mice per group making a total of 24 mice. Based on the behavioral outcomes, 1 μg/paw of BjV was selected as the effective dose for subsequent experiments.

A separate dose–response study was conducted to identify the optimal MaR2 dose. Mice were pretreated with a single intraperitoneal (i.p.) injection of vehicle (100 μL saline) or MaR2 (0.3, 1, or 3 ng/cavity in 100 μL saline), administered 30 min prior to BjV injection. Hyperalgesia was evaluated using thermal and mechanical assays (5 groups with 6 mice per group making a total of 30 mice). The dose of 3 ng/cavity was selected based on maximal efficacy. Static Weight Bearing (SWB) analysis used 3 groups with 6 mice per group making a total of 18 mice. The plantar tissue was collected 7 h post-BjV injection to be analyzed in assays for myeloperoxidase (MPO) activity (3 groups, 12 mice per group, total of 36 mice), Nitroblue Tetrazolium (NBT) and ABTS assays (3 groups, 6 mice per group, total of 18 mice), and ELISA for cytokines (3 groups, 8 mice per group, total of 24 mice) were performed.

In the next set of experiments, an i.p. dose–response curve of BjV (1, 3, or 5 μg/cavity) (4 groups, 8 mice per group, total of 32 mice) was established with sample collection 15 h post-stimulus injection. The dose of 3 μg was selected based on total and differential leukocyte counts. A similar approach was used to define the optimal MaR2 dose (0.1, 1, or 10 ng/cavity) (5 groups, 8 mice per group, total of 40 mice), with 1 ng selected as most effective. Additional analyses included peritoneal erythrocyte counts (3 groups, 8 mice per group, total of 24 mice), reactive oxygen species (ROS) (3 groups, 10 mice per group, total of 30 mice) and nitric oxide (NO) (3 groups, 9 mice per group, total of 27 mice) quantification via fluorescence probes, NBT assay (3 groups, 6 mice per group, total of 18 mice), and *gp91phox* and *iNOS* mRNA expression using RT-qPCR (3 groups, 5 mice per group, total of 15 mice). Supplementary Table S1 presents the number of mice per group per assay.

### 5.3. Test Compounds

We used Maresin 2—(13R,14S)-dihydroxy-(4Z,7Z,9E,11E,16Z,19Z)-docosahexaenoic acid; Cayman Chemicals, Ann Arbor, MI, USA), *Bothrops jararaca* venom (BjV; Laboratory of Herpetology, Butantan Institute, São Paulo, SP, Brazil), saline solution (NaCl 0.9%; Fresenius Kabi Brasil Ltda. Aquiraz, CE, Brazil).

### 5.4. Mechanical Hyperalgesia Assessment

Mechanical hyperalgesia tests were performed by an electronic version of the von Frey filaments, also called anesthesiometer (Insight, Ribeirão Preto, SP, Brazil), as previously described [30]. Mice were placed in acrylic cages with wire grid floors, in a quiet room for at least 1 h before the test for environmental adaptation. The test consists of evoking a right hind paw flexion reflex with a hand-held force transducer adapted with a polypropylene tip. The endpoint was characterized by the removal of the hindlimb followed by clear flinch movements. The value for the response was an average of three measurements. The results are expressed by delta (Δ) withdrawal threshold (basal-time mean measurements were subtracted from mean measurements after BjV injection at indicated time-points.

### 5.5. Thermal Hyperalgesia Assessment

Thermal hyperalgesia was performed by hot plate tests (Insight, Ribeirão Preto, SP, Brazil) at 52 °C ± 1 °C [65]. The reaction time was registered using a conventional chronometer when the mice presented the behaviors of licking or flinching one of the hind paws. A time limit of 15 s of maximum latency was defined as the cut-off. The assessment of thermal hyperalgesia was performed before (baseline T0) and after the BjV injection at indicated time-points. The results were expressed by the thermal threshold in seconds (s).

### 5.6. Static Weight Bearing (SWB)

The Static Weight Bearing (Bioseb, Vitrolles, France) device was used to assess weight distribution imbalance between the inflamed and the non-affected limbs. The animals were individually conditioned in an acrylic device positioned forward, with each mouse’s front paws supported at the front, while their hind legs were supported by a weight measurement sensor (g). The test began the moment the animal was immobilized; then, weight measurements were performed for 10 s. At the end of this period, the weight of the left and right paw was measured, with the right being relative to the stimulus. This test evaluates the weight distribution between a mouse’s hind legs. While an unstimulated animal distributes its weight equally between the two legs, the ratio of weight distribution between the stimulated and unstimulated paw is a measure of the level of discomfort in the stimulated paw. The mean of two measurements were recorded before BjV stimulus (T0) and at indicated time-points after venom administration. Results are presented as right to left paw pressure ratios [30].

### 5.7. Evaluation of Leukocytes Profile

#### 5.7.1. Leukocyte Migration and Differential Cell Counts

*Bothrops jararaca* snake venom induces long-lasting leukocyte migration to the peritoneal cavity [66,67]. To evaluate immune cell recruitment, BjV was injected intraperitoneally, and exudate was collected 15 h later. 5 mL of iced Phosphate-Buffered Saline (PBS) (1x), pH 7.0, with EDTA 2 mM were injected into the peritoneum to obtain recruited leukocytes. A total of 20 μL of exudate was diluted in 180 μL of Turk solution (to lyse erythrocytes), and total leukocyte counts were performed with a Neubauer chamber. Differential leukocyte count was carried out on microscopy slides prepared using a cytocentrifuge (Shandon Cytospin 3, Tokyo, Japan), stained with fast panoptic (Laborclin, Pinhais, Brazil), and analyzed with brightfield microscopy (Olympus CX31RTSF, Tokyo, Japan). Results were expressed as total leukocyte count (cells × 10^6^/cavity) and percentage (%) of polymorph and mononuclear cells.

#### 5.7.2. Myeloperoxidase Activity (MPO)

Mice plantar paw tissue neutrophil migration was evaluated by the colorimetric detection of myeloperoxidases, as previously described [65]. Samples were collected 7 h after intraplantar injection on iced K_2_HPO_4_ (50 mM) buffer (pH 6.0) with 0.5% HTAB. Briefly, samples were homogenized and centrifuged (14,000 rpm × 2 min) and 5 μL of the supernatant was added to 200 μL of phosphate buffer (50 mM), pH 6.0, with 0.167 mg/mL of o-dianisidine dihydrochloride and 0.015% hydrogen peroxide. The absorbance was measured at 600 nm after 20 min. The MPO activity of the sample was compared to a standard curve of neutrophils. Results were presented as MPO activity (number of neutrophils × 10^3^ per milligram of tissue).

### 5.8. Oxidative Stress

#### 5.8.1. Nitroblue Tetrazolium Reduction (NBT) Assay

Superoxide anion production on paw tissue (7 h) homogenate and peritoneal cell (15 h) wash was assessed through NBT assay [68]. A total of 100 μL of tissue homogenate or peritoneal wash was added to 100 μL of NBT 1mg/mL on a 96 well plate for 1 h. Supernatant was removed and precipitated formazan crystals were solubilized adding 120 µL of KOH 2 M and 120 µL of dimethyl sulfoxide (DMSO). NBT reduction was measured at 600nm by spectrophotometry Microplate Spectrophotometer (Multiskan GO, ThermoScientific, Vantaa, Finland). Results from tissue samples were presented as optical density per mg of tissue, whereas results from peritoneal wash were presented as optical density per μg of protein.

#### 5.8.2. Microscopic NBT-Positive Cell Count

Production of superoxide anion was evaluated by the deposition of formazan crystals in the cytoplasm and cellular membrane of leukocytes. The reduction of NBT from a light-yellow dye into a precipitated dark blue crystal (formazan) is observed under light microscopy [68]. For the microscopic assessment of NBT-positive cells, the peritoneal exudate was collected 7 h after the BjV stimulus. A total of 50 μL of peritoneal wash was incubated with 50 μL of NBT (1 mg/mL, Sigma-Aldrich, St. Louis, MO, USA) at room temperature for 30 min. Microscopy slides were prepared using a cytocentrifuge (Shandon Cytospin 3, Tokyo, Japan), stained with fast panoptic (Laborclin, Pinhais, Brazil), and the number of NBT-positive cells was counted under light microscopy (Olympus CX31RTSF, Tokyo, Japan). The values were expressed as a percentage of NBT-positive cells.

#### 5.8.3. Antioxidant Efficiency by ABTS

Oxidative damage resistance was investigated through the efficiency of eliminating free radicals observed by ABTS assay [68]. Plantar paw tissue was collected 7 h after BjV stimulus, homogenized in 500 µL of KCl (1.15%) using a Tissue-Tearor (Biospec 985370), and centrifuged at 1000× *g* for 10 min at 4 °C and the supernatant was used to measure the antioxidant efficiency of MaR2. The solution of ABTS was prepared with 7 mM of ABTS and 2.45 mM of potassium persulfate diluted with phosphate buffer pH 7.4 to an absorbance of 0.7–0.8 in 730 nm was prepared. The supernatant was mixed on ABTS solution, and after 6 min, the absorbance was determined in a 730 nm microplate reader (Multiskan GO, ThermoScientific, Vantaa, Finland). Previously, a standard Trolox curve (0.01 a 20 nmol) was prepared, and the results were expressed as equivalent nmol of Trolox/mg of tissue.

#### 5.8.4. Total Intracellular ROS Detection

Production of intracellular ROS was evaluated using an H2DCFDA probe (ID: D99, H2-DCF, Invitrogen, Waltham, MA, USA) [68]. Peritoneal exudate wash was collected 15 h after i.p. injection of BjV on Facs Buffer (PBS [152 mM], pH 7.0, EDTA [2 mM], and 0.5% Bovine Serum Albumin [BSA]). Samples were centrifuged (1200 rpm, 10 min), supernatant were discarded, and cell pellets were seeded on microscopic glass bottom culture plates using incomplete RPMI medium. Cells were kept in CO_2_ incubator (37 °C, CO_2_ 5%) for 30 min to adhere. Probe H2DCFDA 20 mM (1:200) was added for incubation for another 40 min, plates were washed three times with HBSS, and cells were analyzed by confocal microscopy (TCS SP8, Leica, Mannheim, Germany), with objective of 63X on 488 wavelength fluorescence using Software Leica X (LAS X, Leica, Mannheim, Germany) to obtain images. Analysis was performed by investigators blinded to the experimental groups.

#### 5.8.5. Intracellular Nitric Oxide (NO) Detection

Intracellular nitric oxide levels were assessed using fluorescence microscopy with DAF-FM (D23842, Invitrogen, Waltham, MA, USA) probe [68]. Cells were collected and procedures were carried out as described on item 2.8.5 until the point of probing. For NO detection, DAF-FM (1:500) probe was added, plates were washed three times after 40 min of incubation, and images were obtained by confocal microscopy (TCS SP8, Leica, Mannheim, Germany), with objective of 63X on 488 wavelength fluorescence using Software Leica X (LAS X 5.2.2, Leica, Mannheim, Germany) to obtain images. Analysis was performed by investigators blinded to the experimental groups.

### 5.9. Cytokines Quantitation

Mice plantar paw tissue was collected 7 h after intraplantar injection of BjV and frozen at −80 °C. Briefly, samples were homogenized using an Ultra Turrax^®^ (IKA T10 basic, Vernon Hills, IL, USA) on 500 μL of saline solution 0.9% and centrifuged (3000 rpm × 15 min × 4 °C). Supernatant were collected to determine cytokines TNF-α, IL-1β, and IL-6 levels using ELISA commercial kits following manufacturers’ instructions (DuoSet ELISA Development [R&D Systems, DY410; DY401] and ELISA MAX™ Deluxe Set Mouse IL-6 [Biolegend, 431301], respectively) [68]. Results were presented as pg per mg of tissue.

### 5.10. Erythrocyte Count

Metalloproteinases present in snake venom are known to generate a series of symptoms, mainly related to local or systemic hemorrhage [41,59]. To investigate hemorrhage in the peritoneal cavity after BjV induction, exudate was collected according to the procedures followed on total and differential leukocyte counts assays, but instead of using Tukey solution, 180 μL of Dacie solution, used to lyse leukocytes, was added to 20 μL of peritoneal exudate. Erythrocyte count was performed using a Neubauer chamber. Results were expressed as total erythrocytes × 10^3^ per cavity.

### 5.11. RT-qPCR

Peritoneal exudates were collected 7 h after i.p. injection of BjV. The abdominal cavity was washed once with 5 mL of sterile saline with EDTA 2mM. Cells were lysed (QIAzol Lysis Reagent, QIAGEN, ID: 79306) and total RNA was extracted following manufacturer instructions [59]. RNA purity and concentration were measured by UV spectrophotometry (Multiskan GO Microplate Spectrophotometer, Thermo Scientific, Vantaa, Finland) (260/280 nm) adopting purity cutoff between 1.8 and 2.0. Reverse transcription was carried out using QuantiTect Reverse Transcription Kit (400) (QIAGEN, 205314) and for qPCR, the QuantiNova SYBR Green PCR Kit (QIAGEN, 208056) was used. Readings were performed using StepOnePlusTM Real-Time PCR System (Applied Biosystems ^®®^, Waltham, MA, USA). Relative gene expression was obtained through the comparative method 2−(ΔΔCt). Primers sequences used for this analysis are detailed in Table 1. The constitutive *Gapdh* gene was used to normalize data [65].

### 5.12. Statistical Analysis

Sample size (n = 6–12 animals per group depending on the assay and informed in the figure captions) was determined for each assay using G*Power 3.1. Data were analyzed using the software GraphPad Prism (GraphPad Software, version 9.0.0). Normally distributed data were analyzed with one or two-way ANOVA (according to the presence or absence of multiple time-points) followed by Tukey. Data without normal distribution were analyzed with Kruskal–Wallis non-parametric test followed by Dunn. Detailed information regarding statistical analysis is presented in Supplementary Table S1, which includes information on sample sizes and one- or two-way ANOVA F and *p* values. Results are presented as mean + SEM, ± SD (for parametric data) or median with range (for non-parametric data). Groups were considered statistically different if *p*-value ≤ 0.05.

## Figures and Tables

**Table 1:** Primer sequences used in RT-qPCR.

Gene	Sense	Antisense	Manufacturer and Prime Number
*Gapdh*	5′-GCCCAGAACATCATCCCTGC-3′	5′-GCCTCTCTTGCTCAGTGTCC-3′	Invitrogen Sense: I3932A11 Antisense: 13932A12
Gp91phox (*Cybb* gene)	5′-AGCTATGAGGTGGTGATGTTAGTGG-3′	5′-CACAATATTTGTACCAGACAGACTTGAG-3′	Invitrogen Sense: I2203C11 Antisense: I2203C12
iNOS (*Nos2* gene)	5′-CGAAACGCTTCACTTCCAA-3′	5′-TGAGCCTATATTGCTGTGGCT-3′	Invitrogen Sense: I2203B07 Antisense: I2203B08

## Data Availability

The original contributions presented in this study are included in this article and Supplementary Material. Further inquiries can be directed to the corresponding author.

## Supplementary Materials

The following supporting information can be downloaded at: https://www.mdpi.com/article/10.3390/toxins17080367/s1, Table S1: Statistical information from results shown in Figure 1, Figure 2, Figure 3, Figure 4, Figure 5, Figure 6, Figure 7, Figure 8, Figure 9 and Figure 10.

## Abbreviations

The following abbreviations are used in this manuscript:

ABTS	2,2′-azino-bis(3-ethylbenzothiazoline-6-sulfonic acid
BjV	*Bothrops jararaca* venom
CGRP	Calcitonin gene-related peptide
DMSO	Dimethyl sulfoxide
EDTA	*Ácido etilenodiaminotetracético*
IASP	International Association for the Study of Pain
iNOS	Inducible nitric oxide synthase
HTAB	Hexadecyltrimethylammonium bromide
IL-1β	Interleukin-1β
IL-6	Interleukin-6
I.P.	Intraperitoneal
I.PL.	Intraplantar
KCl	Potassium chloride
K2HPO4	Dipotassium phosphate
LPS	Lipopolysaccharide
MaR1	Maresin 1
MaR2	Maresin 2
MPO	Myeloperoxidase
NBT	Nitroblue tetrazolium
NFκB	Nuclear factor kappa-B
NLRP3	Nucleotide-binding oligomerization domain (NOD)-like receptor pyrin domain containing 3
NO	Nitric oxide
O_2_^•−^	Superoxide anion
PBS	Phosphate-buffered saline
PMN	Polymorphonuclear leukocyte
RNS	Reactive nitrogen species
ROS	Reactive oxygen species
SPM	Specialized pro-resolution lipid mediators
SWB	Static weight bearing
TRPA1	Transient receptor potential ankyrin 1
TRPV1	Transient receptor potential vanilloid 1
TNF-α	Tumor necrosis factor alpha

## References

[1] da Saúde M. (2022). Boletim Epidemiológico Vol. 53-N^o^ 36.

[2] Ribeiro L.A., Jorge M.T. (1997). Bites by snakes in the genus Bothrops: A series of 3139 cases. Rev. Soc. Bras. Med. Trop..

[3] Ferraz C.R., Carvalho T.T., Fattori V., Saraiva-Santos T., Pinho-Ribeiro F.A., Borghi S.M., Manchope M.F., Zaninelli T.H., Cunha T.M., Casagrande R. (2021). Jararhagin, a Snake Venom Metalloproteinase, Induces Mechanical Hyperalgesia in Mice with the Neuroinflammatory Contribution of Spinal Cord Microglia and Astrocytes. Int. J. Biol. Macromol..

[4] Ferraz C.R., Calixto-Campos C., Manchope M.F., Casagrande R., Clissa P.B., Baldo C., Verri W.A. (2015). Jararhagin-Induced Mechanical Hyperalgesia Depends on TNF-α, IL-1β and NFκB in Mice. Toxicon.

[5] Clissa P.B., Laing G.D., Theakston R.D.G., Mota I., Taylor M.J., Moura-da-Silva A.M. (2001). The Effect of Jararhagin, a Metalloproteinase from *Bothrops Jararaca* Venom, on pro-Inflammatory Cytokines Released by Murine Peritoneal Adherent Cells. Toxicon.

[6] Gutiérrez J.M., Lomonte B. (1995). Phospholipase A2 Myotoxins from Bothrops Snake Venoms. Toxicon.

[7] Bjarnason J.B., Fox J.W. (1994). Hemorrhagic Metalloproteinases from Snake Venoms. Pharmacol. Ther..

[8] Petricevich V.L., Teixeira C.F., Tambourgi D.V., Gutiérrez J.M. (2000). Increments in Serum Cytokine and Nitric Oxide Levels in Mice Injected with Bothrops Asper and *Bothrops jararaca* Snake Venoms. Toxicon.

[9] Júnior F.A.N., Jorge A.R.C., Marinho A.D., Silveira J.A.D.M., Alves N.T.Q., Costa P.H.S., Silva P.L.B.E., Chaves-Filho A.J.M., Lima D.B., Sampaio T.L. (2019). Bothrops Alternatus Snake Venom Induces Cytokine Expression and Oxidative Stress on Renal Function. Curr. Top. Med. Chem..

[10] Redza-Dutordoir M., Averill-Bates D.A. (2016). Activation of Apoptosis Signalling Pathways by Reactive Oxygen Species. Biochim. Biophys. Acta.

[11] Picolo G., Chacur M., Gutiérrez J.M., Teixeira C.F.P., Cury Y. (2002). Evaluation of Antivenoms in the Neutralization of Hyperalgesia and Edema Induced by *Bothrops jararaca* and Bothrops Asper Snake Venoms. Brazilian J. Med. Biol. Res. Rev. Bras. Pesqui. Medicas e Biol..

[12] Muniz E.G., Maria W.S., Estevão-Costa M.I., Buhrnheim P., Chávez-Olórtegui C. (2000). Neutralizing Potency of Horse Antibothropic Brazilian Antivenom against Bothrops Snake Venoms from the Amazonian Rain Forest. Toxicon.

[13] Dias da Silva W., De Andrade S.A., Megale Â.A.A., De Souza D.A., Sant’Anna O.A., Magnoli F.C., Guidolin F.R., Godoi K.S., Saladini L.Y., Spencer P.J. (2022). Antibodies as Snakebite Antivenoms: Past and Future. Toxins.

[14] Kondo F.V., Cabrera W.H.K., Ribeiro O.G., De Franco M., Jensen J.R., Picolo G., Sant’Anna M.B., Spadafora-Ferreira M., Borrego A., Ibañez O.M. (2021). Pain and Cellular Migration Induced by *Bothrops jararaca* Venom in Mice Selected for an Acute Inflammatory Response: Involvement of Mast Cells. Front. Immunol..

[15] Rezende R.M., França D.S., Menezes G.B., dos Reis W.G.P., Bakhle Y.S., Francischi J.N. (2008). Different Mechanisms Underlie the Analgesic Actions of Paracetamol and Dipyrone in a Rat Model of Inflammatory Pain. Br. J. Pharmacol..

[16] Ghosh M., Acharyya A., Bhattacharya P., Chakrabortty S. (2022). Role of steroid on management of limb swelling and local pain in haematotoxic snake bite. J. Family Med. Prim. Care..

[17] Stormholt E.R., Steiness J., Derby C.B., Larsen M.E., Maagaard M., Mathiesen O. (2023). Glucocorticoids Added to Paracetamol and NSAIDs for Post-Operative Pain: A Systematic Review with Meta-Analysis and Trial Sequential Analysis. Acta Anaesthesiol. Scand.

[18] Bonilla-Aldana D.K., Bonilla-Aldana J.L., Ulloque-Badaracco J.R., Al-Kassab-Córdova A., Hernandez-Bustamante E.A., Alarcon-Braga E.A., Siddiq A., Benites-Zapata V.A., Rodriguez-Morales A.J., Luna C. (2024). Snakebite-Associated Infections: A Systematic Review and Meta-Analysis. Am. J. Trop. Med. Hyg..

[19] Fetrow K.O. (1989). The Management of Pain in Orthopaedics. Clin. J. Pain.

[20] Buckley C.D., Gilroy D.W., Serhan C.N. (2014). Proresolving Lipid Mediators and Mechanisms in the Resolution of Acute Inflammation. Immunity.

[21] Serhan C.N., Chiang N. (2013). Resolution Phase Lipid Mediators of Inflammation: Agonists of Resolution. Curr. Opin. Pharmacol..

[22] Zaninelli T.H., Fattori V., Verri W.A.J. (2021). Harnessing Inflammation Resolution in Arthritis: Current Understanding of Specialized Pro-Resolving Lipid Mediators’ Contribution to Arthritis Physiopathology and Future Perspectives. Front. Physiol..

[23] Fattori V., Zaninelli T.H., Rasquel-Oliveira F.S., Casagrande R., Verri W.A. (2020). Specialized Pro-Resolving Lipid Mediators: A New Class of Non-Immunosuppressive and Non-Opioid Analgesic Drugs. Pharmacol. Res..

[24] Rasquel-Oliveira F.S., Silva M.D.V.D., Martelossi-Cebinelli G., Fattori V., Casagrande R., Verri W.A.J. (2023). Specialized Pro-Resolving Lipid Mediators: Endogenous Roles and Pharmacological Activities in Infections. Molecules.

[25] Ji R.-R. (2023). Specialized Pro-Resolving Mediators as Resolution Pharmacology for the Control of Pain and Itch. Annu. Rev. Pharmacol. Toxicol..

[26] Fattori V., Pinho-Ribeiro F.A., Staurengo-Ferrari L., Borghi S.M., Rossaneis A.C., Casagrande R., Verri W.A., Verri W.A. (2019). The Specialised Pro-Resolving Lipid Mediator Maresin 1 Reduces Inflammatory Pain with a Long-Lasting Analgesic Effect. Br. J. Pharmacol..

[27] Deng B., Wang C.-W., Arnardottir H.H., Li Y., Cheng C.-Y.C., Dalli J., Serhan C.N. (2014). Maresin Biosynthesis and Identification of Maresin 2, a New Anti-Inflammatory and pro-Resolving Mediator from Human Macrophages. PLoS ONE.

[28] Miranda J., Brazil J.C., Morris A.H., Parkos C.A., Quiros M., Nusrat A. (2023). Maresin-2 Promotes Mucosal Repair and Has Therapeutic Potential When Encapsulated in Thermostable Nanoparticles. Proc. Natl. Acad. Sci. USA.

[29] Tozzi O.N., Jiacomini I.G., Bastos T.S.B., Nicolazzi L.H.C.N., Dos Santos Luz R.B., Paredes L.C., Gonçalves L.E., Lima M.H.S., Verri W.A.J., Camara N.O.S. (2022). Evaluation of the Effects of Loxosceles Intermedia’s Venom in Zebrafish. Toxicol. Rep..

[30] Fattori V., Zaninelli T.H., Ferraz C.R., Brasil-Silva L., Borghi S.M., Cunha J.M., Chichorro J.G., Casagrande R., Verri W.A.J. (2022). Maresin 2 Is an Analgesic Specialized Pro-Resolution Lipid Mediator in Mice by Inhibiting Neutrophil and Monocyte Recruitment, Nociceptor Neuron TRPV1 and TRPA1 Activation, and CGRP Release. Neuropharmacology.

[31] Lopes R.V., Baggio D.F., Ferraz C.R., Bertozzi M.M., Saraiva-Santos T., Verri Junior W.A., Chichorro J.G. (2023). Maresin-2 Inhibits Inflammatory and Neuropathic Trigeminal Pain and Reduces Neuronal Activation in the Trigeminal Ganglion. Curr. Res. Neurobiol..

[32] Serhan C.N., Levy B.D. (2018). Resolvins in Inflammation: Emergence of the pro-Resolving Superfamily of Mediators. J. Clin. Investig..

[33] Kay J., Thadhani E., Samson L., Engelward B. (2019). Inflammation-Induced DNA Damage, Mutations and Cancer. DNA Repair.

[34] Klebanoff S.J. (1999). Myeloperoxidase. Proc. Assoc. Am. Physicians.

[35] Serhan C.N. (2014). Pro-Resolving Lipid Mediators Are Leads for Resolution Physiology. Nature.

[36] Escalante T., Núñez J., Moura da Silva A.M., Rucavado A., Theakston R.D.G., Gutiérrez J.M. (2003). Pulmonary Hemorrhage Induced by Jararhagin, a Metalloproteinase from Bothrops Jararaca Snake Venom. Toxicol. Appl. Pharmacol..

[37] Moura-da-Silva A.M., Ramos O.H.P., Baldo C., Niland S., Hansen U., Ventura J.S., Furlan S., Butera D., Della-Casa M.S., Tanjoni I. (2008). Collagen Binding Is a Key Factor for the Hemorrhagic Activity of Snake Venom Metalloproteinases. Biochimie.

[38] Kamiguti A.S., Hay C.R., Theakston R.D., Zuzel M. (1996). Insights into the Mechanism of Haemorrhage Caused by Snake Venom Metalloproteinases. Toxicon.

[39] Zuliani J.P., Soares A.M., Gutiérrez J.M. (2020). Polymorphonuclear neutrophil leukocytes in snakebite envenoming. Toxicon.

[40] Yu C.-X., Shi Z.-A., Ou G.-C., Chen X.-J., Liu Q., Zeng D., Nie X.-J., Chen J.-J. (2022). Maresin-2 Alleviates Allergic Airway Inflammation in Mice by Inhibiting the Activation of NLRP3 Inflammasome, Th2 Type Immune Response and Oxidative Stress. Mol. Immunol..

[41] Silva G.M., Berto D.H., Lima C.A., Waitman K.B., Lima C.F.G., Prezoto B.C., Vieira M.L., Rocha M.M.T., Gonçalves L.R.C., Andrade S.A. (2021). Synergistic Effect of Serine Protease Inhibitors and a Bothropic Antivenom in Reducing Local Hemorrhage and Coagulopathy Caused by *Bothrops jararaca* Venom. Toxicon.

[42] Fattori V., Hohmann M.S., Rossaneis A.C., Pinho-Ribeiro F.A., Verri W.A. (2016). Capsaicin: Current Understanding of Its Mechanisms and Therapy of Pain and Other Pre-Clinical and Clinical Uses. Molecules.

[43] Verri W.A., Cunha T.M., Parada C.A., Wei X.Q., Ferreira S.H., Liew F.Y., Cunha F.Q., Verri W.A., Cunha T.M., Parada C.A. (2006). IL-15 Mediates Immune Inflammatory Hypernociception by Triggering a Sequential Release of IFN-Gamma, Endothelin, and Prostaglandin. Proc. Natl. Acad. Sci. USA.

[44] Varela M.L., Mogildea M., Moreno I., Lopes A. (2018). Acute Inflammation and Metabolism. Inflammation.

[45] Teixeira C.F.P., Chaves F., Zamunér S.R., Fernandes C.M., Zuliani J.P., Cruz-Hofling M.A., Fernandes I., Gutiérrez J.M. (2005). Effects of Neutrophil Depletion in the Local Pathological Alterations and Muscle Regeneration in Mice Injected with *Bothrops jararaca* Snake Venom. Int. J. Exp. Pathol..

[46] Cedro R.C.A., Menaldo D.L., Costa T.R., Zoccal K.F., Sartim M.A., Santos-Filho N.A., Faccioli L.H., Sampaio S.V. (2018). Cytotoxic and Inflammatory Potential of a Phospholipase A2 from *Bothrops jararaca* Snake Venom. J. Venom. Anim. Toxins Incl. Trop. Dis..

[47] Farsky S.H.P., Borelli P., Fock R.A., Proto S.Z., Ferreira J.M.C., Melo S.B. (2004). V Chronic Blockade of Nitric Oxide Biosynthesis in Rats: Effect on Leukocyte Endothelial Interaction and on Leukocyte Recruitment. Inflamm. Res..

[48] Farsky S.H., Walber J., Costa-Cruz M., Cury Y., Teixeira C.F. (1997). Leukocyte Response Induced by *Bothrops jararaca* Crude Venom: In Vivo and in Vitro Studies. Toxicon.

[49] Kolaczkowska E., Kubes P. (2013). Neutrophil Recruitment and Function in Health and Inflammation. Nat. Rev. Immunol..

[50] Sikora J.P., Karawani J., Sobczak J. (2023). Neutrophils and the Systemic Inflammatory Response Syndrome (SIRS). Int. J. Mol. Sci..

[51] El Kebir D., József L., Pan W., Filep J. (2008). Myeloperoxidase delays neutrophil apoptosis through CD11b/CD18 integrins and prolongs inflammation. Circ. Res..

[52] Lau D., Mollnau H., Eiserich J.P., Freeman B.A., Daiber A., Gehling U.M., Brümmer J., Rudolph V., Münzel T., Heitzer T. (2005). Myeloperoxidase Mediates Neutrophil Activation by Association with CD11b/CD18 Integrins. Proc. Natl. Acad. Sci. USA.

[53] Carr A.C., Winterbourn C.C. (1997). Oxidation of neutrophil glutathione and protein thiols by myeloperoxidase-derived hypochlorous acid. Biochem. J..

[54] Rutgers A., Heeringa P., Giesen J.E.H.M., Theunissen R.T., Jacobs H., Tervaert J.W.C. (2003). Neutrophil Myeloperoxidase Activity and the Influence of Two Single-Nucleotide Promoter Polymorphisms. Br. J. Haematol..

[55] Park M.D., Silvin A., Ginhoux F., Merad M. (2022). Macrophages in Health and Disease. Cell.

[56] Sugiyama S., Okada Y., Sukhova G.K., Virmani R., Heinecke J.W., Libby P. (2001). Macrophage Myeloperoxidase Regulation by Granulocyte Macrophage Colony-Stimulating Factor in Human Atherosclerosis and Implications in Acute Coronary Syndromes. Am. J. Pathol..

[57] Aratani Y. (2018). Myeloperoxidase: Its Role for Host Defense, Inflammation, and Neutrophil Function. Arch. Biochem. Biophys..

[58] Zychar B.C., Dale C.S., Demarchi D.S., Gonçalves L.R.C. (2010). Contribution of Metalloproteases, Serine Proteases and Phospholipases A2 to the Inflammatory Reaction Induced by *Bothrops Jararaca* Crude Venom in Mice. Toxicon.

[59] Abad Ribeiro A.B., Santoro M.L., Duarte M.R., Virgulino C.C., de Oliveira G.S.S., França F.O.D.S. (2024). Hemoperitoneum after a Bothrops Snakebite: Case Report. Toxicon.

[60] Förstermann U., Sessa W.C. (2012). Nitric Oxide Synthases: Regulation and Function. Eur. Heart J..

[61] Zamuner S.R., Gutiérrez J.M., Muscará M.N., Teixeira S.A., Teixeira C.F. (2001). Bothrops Asper and *Bothrops jararaca* Snake Venoms Trigger Microbicidal Functions of Peritoneal Leukocytes in Vivo. Toxicon.

[62] Serhan C.N., Dalli J., Karamnov S., Choi A., Park C., Xu Z., Ji R., Zhu M., Petasis N.A. (2012). Macrophage Proresolving Mediator Maresin 1 Stimulates Tissue Regeneration and Controls Pain. FASEB J..

[63] Maximiano T.K.E., Carneiro J.A., Fattori V., Verri W.A. (2024). TRPV1: Receptor Structure, Activation, Modulation and Role in Neuro-Immune Interactions and Pain. Cell Calcium.

[64] Almeida A.S.D., Bernardes L.D.B., Trevisan G. (2021). TRP Channels in Cancer Pain. Eur. J. Pharmacol..

[65] Lourenco-Gonzalez Y., Fattori V., Domiciano T.P., Rossaneis A.C., Borghi S.M., Zaninelli T.H., Bernardy C.C.F., Alves-Filho J.C., Cunha T.M., Cunha F.Q. (2019). Repurposing of the Nootropic Drug Vinpocetine as an Analgesic and Anti-Inflammatory Agent: Evidence in a Mouse Model of Superoxide Anion-Triggered Inflammation. Mediat. Inflamm..

[66] Zamuner S.R., Teixeira C.F.P. (2002). Cell Adhesion Molecules Involved in the Leukocyte Recruitment Induced by Venom of the Snake Bothrops Jararaca. Mediat. Inflamm..

[67] da Silva Fernandes Ribas A., de Godoi K.S., Sant’Anna S.S., da Rocha M.M.T., da Silva W.D. (2025). Release of Cytokines in the Peritoneal Fluid of C57BL/6 Mice After Bothrops Jararaca and Bothrops Atrox Venom Injection. Toxins.

[68] Fattori V., Rasquel-Oliveira F.S., Artero N.A., Ferraz C.R., Borghi S.M., Casagrande R., Verri W.A. (2020). Diosmin Treats Lipopolysaccharide-Induced Inflammatory Pain and Peritonitis by Blocking NF-ΚB Activation in Mice. J. Nat. Prod..

