# Supplementary material for: Maresin 2, a Specialized Pro-Resolution Lipid Mediator, Reduces Pain and Inflammation Induced by Bothrops jararaca Venom in Mice
Source: Toxins (Basel). 2025 Jul 25;17(8):367. doi: 10.3390/toxins17080367 (PMC12389977; doi:10.3390/toxins17080367)
Supplement: Supplementary file 1 [file toxins-17-00367-s001.zip › toxins-3597110-supplementary.pdf]

Supplementary Materials

Table S1. Statistical information from results shown in Figures 1-10.

Statistical test	Post-test	F (DFn, DFd) value	P value	n per group
Two-way ANOVA				
Figure 1**b**	Tukey	Interaction (Time x Dose): F (21, 140) = 11.13<br>Time: F (4,818, 96,36) = 58.55<br>Dose: F (3, 20) = 42.18	P < 0.0001	6
Figure 1**c**	Tukey	Interaction (Time x Dose): F (24, 160) = 2.270<br>Time: F (5,406, 108,1) = 6.681<br>Dose: F (3, 20) = 7.427	P=0.0014	6
Figure 2**b**	Tukey	Interaction (Time x Dose): F (28, 175) = 6.773<br>Time: F (5,484, 137,1) = 17.71<br>Dose: F (4, 25) = 67.16	P<0.0001	6
Figure 2**c**	Tukey	Interaction (Time x Dose): F (32, 200) = 1.755<br>Time: F (5,584, 139,6) = 11.94<br>Dose: F (4, 25) = 9.268	P=0.0110	6
Figure 2**d**	Tukey	Interaction (Time x Dose): F (16, 135) = 3.543<br>Time: F (8, 135) = 8.124<br>Dose: F (2, 135) = 16.24	P<0.0001	6
One-way ANOVA				
Figure 3**a**	Tukey	F (2, 33) = 73.38	P<0.0001	12
Figure 3**b**	Tukey	F (2, 15) = 13.53	P=0.0004	6
Figure 3**c**	Tukey	F (2, 15) = 7.151	P=0.0066	6
Figure 4**a**	Tukey	F (2, 21) = 58.93	P<0.0001	8
Figure 4**b**	Tukey	F (2, 21) = 12.84	P=0.0002	8
Figure 4**c**	Tukey	F (2, 21) = 15.65	P<0.0001	8
Figure 5**b**	Tukey	F (3, 28) = 23.53	P<0.0001	8
Figure 5**c**	Tukey	F (3, 28) = 8.683	P=0.0003	8
Figure 5**d**	Tukey	F (3, 28) = 13.98	P<0.0001	8
Figure 6**b**	Tukey	F (4, 35) = 21.69	P<0.0001	8
Figure 6**c**	Tukey	F (4, 35) = 12.52	P<0.0001	8
Figure 6**d**	Kruskal-Wallis		P<0.0001	8
Figure 7	Tukey	F (2, 21) = 32.42	P<0.0001	8
Figure 8**a**	Tukey	F (2, 15) = 42.61	P<0.0001	6
Figure 8**b**	Tukey	F (2, 15) = 61.05	P<0.0001	6
Figure 9**b**	Tukey	F (2, 27) = 10.24	P=0.0005	10
Figure 9**c**	Tukey	F (2, 12) = 7.846	P=0.0066	5
Figure 10**b**	Tukey	F (2, 24) = 12.12	P=0.0002	10
Figure 10**c**	Tukey	F (2, 12) = 8.146	P=0.0058	5
